# Parasites of Selected Freshwater Snails in the Eastern Murray Darling Basin, Australia

**DOI:** 10.3390/ijerph19127236

**Published:** 2022-06-13

**Authors:** Diane P. Barton, Xiaocheng Zhu, Alara Nuhoglu, Luke Pearce, Matthew McLellan, Shokoofeh Shamsi

**Affiliations:** 1 School of Agricultural, Environmental and Veterinary Sciences, Charles Sturt University, Wagga Wagga, NSW 2678, Australia; dibarton@csu.edu.au (D.P.B.); xiaocheng.zhu@dpi.nsw.gov.au (X.Z.); alaranuhoglu@gmail.com (A.N.); 2 NSW Department of Primary Industries, Wagga Wagga Agricultural Institute, Wagga Wagga, NSW 2650, Australia; 3 NSW Department of Primary Industries, Fisheries, Habitat & Threatened Species Unit, Freshwater Environment Branch, Albury, NSW 2640, Australia; luke.pearce@dpi.nsw.gov.au; 4 Fisheries and Aquaculture Management, NSW Department of Primary Industries, Narrandera Fisheries Centre, Narrandera, NSW 2700, Australia; matthew.mclellan@dpi.nsw.gov.au

**Keywords:** Trematoda, parasites, freshwater, snails, Murray Darling Basin, life cycle, environment health, invertebrates

## Abstract

Aquatic snails serve an important role in the ecosystem. They also play an essential role in the life cycle of many parasites as hosts and may pose risks to animal and human health. In Australia, the role of snails in the transmission of parasites of livestock is well studied. However, despite the country’s unique biodiversity and wildlife, little is known about the role of snails in the transmission and survival of parasites in other ecosystems, including aquatic and aquaculture systems. This study aimed to determine the occurrence of parasites in freshwater snails in the eastern Murray Darling Basin. A total of 275 snails were collected from various localities, including aquaculture fishery ponds and natural creeks during the summer and autumn months in the southern hemisphere. Three different species of freshwater snails, all common to the area, were found, including *Bullastra lessoni* (*n* = 11), *Isidorella hainesii* (*n* = 157), and *Haitia acuta* (*n* = 107), of which 9.1%, 1.3%, and 4.7%, respectively, were found to be harboring various developmental stages of Trematoda. No other parasite was found in the examined snails. Parasites were identified as *Choanocotyle hobbsi*, *Plagiorchis* sp. and *Petasiger* sp. based on the sequences of their ITS2, 18S, and 28S ribosomal DNA region. Herein, we report a native parasite *Choanocotyle hobbsi* in an introduced snail, *Haitia acuta*, from both natural and aquaculture ponds. As there are no genetic sequences for adult specimens of *Petasiger* spp. and *Plagiorchis* spp. collected in Australia for comparison, whether the specimens collected in this study are the larval stage of one of the previously described species or are a new, undescribed species cannot yet be determined. Our results also suggest snails collected from aquaculture ponds may be infected with considerably more parasites.

## 1. Introduction

Aquatic snails form a significant part of any ecosystem and are important in maintaining the balance of nature in this environment [1,2,3]. For example, because they are on the lower trophic levels of the food web, they are an important food source for many aquatic and aquatic-associated animals (from insects to lizards and snakes, fish, birds, and mammals) [1,2]. Additionally, due to their sensitivity to certain chemicals, aquatic snails can be used as environmental and water quality indicators. Unfortunately, several native freshwater snails in Australia are threatened [4], which is worrisome, considering the important role freshwater snails play in aquatic food webs.

In Australia, almost 500 species of freshwater snails are endemic, with many vulnerable to a wide range of threats, such as introduced species and damage to their habitats [5,6]. There are also over 65 terrestrial and freshwater snails and slugs introduced to Australia [5].

Research on the biology, diseases, and parasites of Australian freshwater snails is scarce. Most of the well-known Australian freshwater snails are only recognized for their important role in the transmission of parasites in agriculture and aquaculture systems and in human health. For example, there is more knowledge about *Lymnaea* spp. due to their role as an intermediate host of liver fluke, *Fasciola hepatica*, a zoonotic trematode infecting herbivores including cattle and sheep [7,8,9], but little is known about those snails that might be intermediate hosts for parasites of wildlife or freshwater animals in Australia.

Knowing which parasites are being transmitted by snails in freshwater systems and the role that introduced snail species may have on the dynamics of parasites through the introduction of exotic parasites and their role as intermediate hosts for native parasites is important to establish biosecurity measures for the growing aquaculture industry in the region, as well as for agriculture, wildlife biodiversity, and human health.

One of the highly diverse regions in Australia is the Murrumbidgee River catchment, located in New South Wales and the Australian Capital Territory. The catchment is home to many wetlands and riverine environments, supports a complex range of natural ecosystems, and has many significant wetland habitats of international ecological importance.

Of the common snails found in the Murrumbidgee River catchment is *Isidorella hainesii* (Tryon, 1866), a native freshwater snail belonging to the family Planorbidae. This snail is commonly found on aquatic vegetation in ponds, billabongs, swamps, and sluggish streams and rivers in the southeastern part of Australia. The taxonomy of *I. hainesii* requires revision [10]. *Bullastra lessoni* (Deshayes, 1830) is another native species belonging to the family Lymnaeidae, which is distributed throughout southern Australia [10]. It is found among water weeds and similar substrates in dams, ponds, billabongs, sluggish rivers, and streams [10]. Another common freshwater snail in eastern Australia is *Haitia acuta* (Draparnaud, 1805), also known as *Physa acuta*, and *Physella acuta*, which is a globally invasive freshwater snail [11]. It is commonly found in Australian inland waters [10]. Taylor [12] transferred *Physella acuta* to the genus *Haitia*, and this has been followed by Ponder et al. [10] in the key for Australian freshwater mollusks.

This study aimed to determine the occurrence of parasites in freshwater snails in the Murrumbidgee catchment area.

## 2. Materials and Methods

### 2.1. Sample Collection

**Figure 1:**
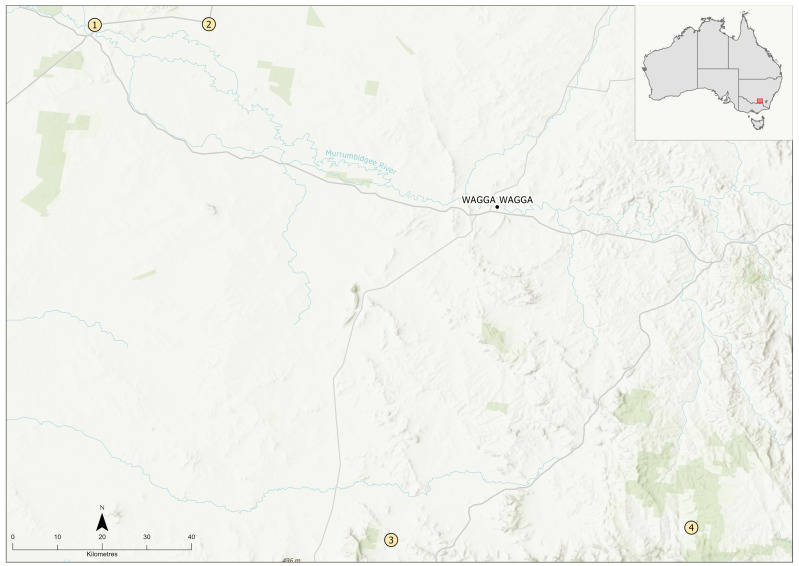
Approximate locations for the collection of snails in the present study: (1) Narrandera; (2) Grong Grong; (3) Mountain Creek; (4) Coppabella Creek, all in New South Wales, Australia. Scale bar represents 20 km. Localities 1 and 2 were a golden perch aquaculture pond with soil bottom, frequented by cormorants, ducks, and egrets. Other life found at the bottom of ponds included yabbies and shrimp. Small bivalves, dipteran insects, and water scorpions were also found in Locality 2. A combination of bore water and river water (Murrumbidgee River) was used for ponds. Locality 3 was a creek flowing through a pine plantation with feral deer, feral pigs, and many native herbivores (kangaroos, wallabies, wombats) but no livestock in the collection area. Cattle and goats were present on properties upstream. Snails were among floating pondweed *Potamogeton tricarinatus.* Other life found among snails included leeches and dragonfly larvae. Locality 4 was a creek flowing through a cattle and sheep property. Dry ewes were in the paddock 2 weeks before collection. Snails were among water ribbons *Vallisneria gigantea* and water couch *Paspalum paspalodes*. The pond was frequented by cormorants, ducks, egrets, and pelicans. Other life found at the bottom of ponds included yabbies, shrimp, small bivalves, water scorpions, and dipteran insects.

A total of 275 snails were collected from various localities, as shown in Figure 1. The collection localities were a combination of aquaculture fishery ponds (locations 1 and 2) and natural creeks (locations 3 and 4). The collection took place during summer and autumn months in the southern hemisphere (February–April 2019). The snails were collected in large specimen jars, approximately half-full of water, and were transported to the Parasitology Laboratory of Charles Sturt University. The snails were identified using Ponder (2020), and all of them were examined by autopsy as described previously [13]. Some parasite specimens were preserved in 70% ethanol for molecular work, and some were mounted permanently in glycerin jelly.

### 2.2. Morphology of Parasites

Slide-mounted specimens were examined by light microscopy. Measurements of total length (TotL), body length (BL), body width (BW), tail length (TL), tail width (TW), tail width with fins (TWF), oral sucker diameter (OS), and ventral sucker diameter (VS) were taken. The numbers of collar spines were counted. Illustrations were created using a microscope equipped with a drawing tube. All measurements are given in micrometers, unless otherwise stated. Mean measurements are specified, followed by the range in parentheses. Photos were taken using a 9 MP Microscope Digital Camera (AmScope Model MU900).

### 2.3. Molecular Diagnostics of Parasites

Single cercaria, redia, or sporocysts were placed in individual Eppendorf tubes and stored at −20 °C until DNA extraction. The samples did not need to be cut, as they were extremely small (<1 mm), and there were many available samples. DNA extraction was completed using the QIAGEN DNeasy Blood and Tissue Kit, following the manufacturer’s instructions. The ITS2, 18S, and 28S regions were amplified using primers and reagents described in Shamsi et al. [13] with the following conditions for all primers and regions: initial denaturation at 95 °C for 2 min; 40 cycles of denaturation (95 °C), annealing (58 °C for both primer pairs), and extension (72 °C) for 30, 30, and 45 s, respectively, followed by a final extension at 72 °C for 10 min. PCR products were Sanger sequenced using the same primer at the Australian Genome Research Facility (Brisbane). Sequences obtained from this study were deposited in the GenBank with accession numbers OM305031-OM305042 (28S region), OM305043-OM305054 (18S region), and OM305095-OM305107 (ITS region).

The sequences were aligned using BioEdit [14]. Primer sequences were removed from analysis. ITS2 sequences of closely related taxa were obtained from GenBank for phylogenetic analyses (Table 1). Where possible, we used sequences obtained from adult specimens associated with morphologically well-identified specimens and peer-reviewed published works. Alignments for ITS2, 28S, and 18S for group A and morphotype B were 1275, 1269, and 1777 bp, respectively. For morphotype C, the alignments of the same regions were 1523, 1225, and 1754, respectively. Descriptions of the groups/morphotypes are provided in the Results section. Alignment gaps were excluded for analyses. Pairwise genetic distances were calculated using MEGA X [15]. The GTR + G, GTR + I + G, and HKY + I models were selected for ITS2, 28S, and 18S regions, respectively, as best fit evolutionary models as inferred by the jModelTest 2 [16]. *Brachycladium goliath* (KR703279) was used as an outgroup for *Choanocotyle* and *Plagiorchis* sp. phylogenetic analyses, as it belongs to the same suborder Xiphidiata but different superfamily. *Philophthalmus gralli* (JX121229 and JQ627832) were used as an outgroup for *Petasiger* sp. phylogenetic analyses, as it belongs to the same superfamily but different family. The phylogeny of selected sequences was calculated using MrBayes 3.2 [17] for 3,000,000 generations for each gene region, with other parameters set as default, until the average standard deviation was lower than 0.005. The first 50% of runs from the Markov chain Monte Carlo algorithm were discarded as burn-in. The tree was visualized using Figtree v 1.4.3 [18].

## 3. Results

Three different species of freshwater snails were found. They are all common to the area. They were found to belong to three distinct families—family Lymnaeidae (*Bullastra lessoni* (*n* = 11)), family Planorbidae (*Isidorella hainesii* (*n* = 157)), and family Physidae (*Haitia acuta* (*n* = 107)). The latter species is an introduced species, which is considered invasive in Australia. Not all snails were infected with parasites. Various developmental stages of Trematoda, including sporocysts, cercariae, and metacercariae, were found in the infected snails. The highest infection rate (9.1%) was observed among *Bullastra lesson*; however, only 11 specimens were available in the present study. Therefore, this infection rate should be viewed with caution. Of the other two species of snails examined herein, *Haitia acuta* and *Isidorella hainesii*, 4.7% and 1.3%, respectively, were found to be infected with Trematoda parasite. No other parasite groups apart from trematodes were found in the examined snails. No mixed infection was observed. Details of the parasites found in different localities and hosts are provided in Table 2.

**Figure 2:**
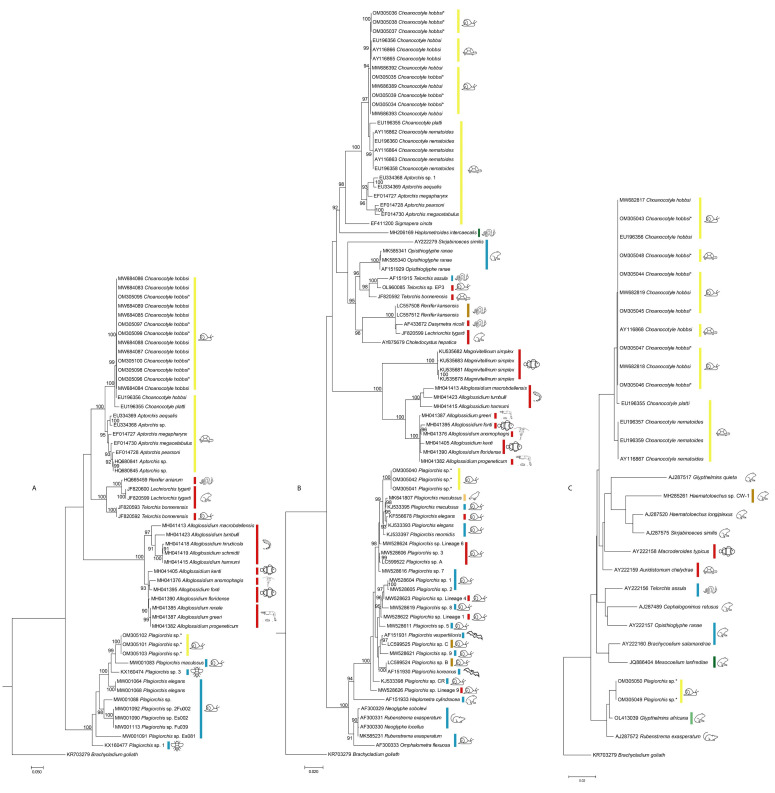
Phylogenetic trees showing the relationship between group A (GenBank accession numbers: OM305040-OM305042, OM305049-OM305050, and OM305101-OM305103) and B (GenBank accession numbers: OM305095-OM305100, OM305034-OM305039, and OM305043-OM305048) in the present study (indicated with *) with closely related taxa in GenBank for (**A**) ITS2, (**B**) 28S, and (**C**) 18S. Geographical area of collection of specimen indicated by a colored bar (red, North America (USA and Mexico); blue, Europe; yellow, Australia; green, Brazil; brown, Japan and China; light brown, Pakistan; light green, Rwanda). The host groups that the parasite was recovered from are shown as icons (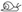, snails; 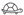, turtles; 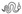, snakes; 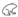, frogs and toads; 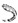, leeches; 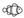, fishes; 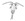, *Daphnia*; 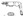, freshwater prawns; 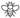, insects; 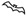, bats; 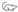, mammals other than bats; 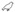, swallow). The hosts are those listed in Table 1 and include hosts from which parasites/sequences were obtained. Some of these hosts are intermediate/paratenic and some are definitive hosts.

The parasites found were all at the larval stage and could not be identified to the species level. Therefore, similar morphotypes were classified into different groups, designated as A to C (Table 2). Cercaria classified as group A did not have any distinguishing characteristics; no morphological description could be performed, as all cercaria found were not fully developed. This is possibly due to the cercaria not emerging from the snail but being removed by dissection. They were identified to the genus *Plagiorchis* based on their sequence data (Figure 2A–C). Sequences from this study were grouped with sequences of *Plagiorchis* spp., primarily from cercarial stages, from throughout Europe for both ITS2 (Figure 2A) and 28S (Figure 2B). For 18S sequences (Figure 2C), however, a lack of available sequences of *Plagiorchis* spp. placed the sequences from this study in a group with specimens of related genera collected from insectivorous hosts (frog, shrew) (see also Table 1).

Group B was found to morphologically and genetically match *Choanocotyle hobbsi* as described in Shamsi, Nuhoglu, Zhu, and Barton [12] (Figure 2A–C) and is referred to as morphotype B in this paper.

**Figure 3:**
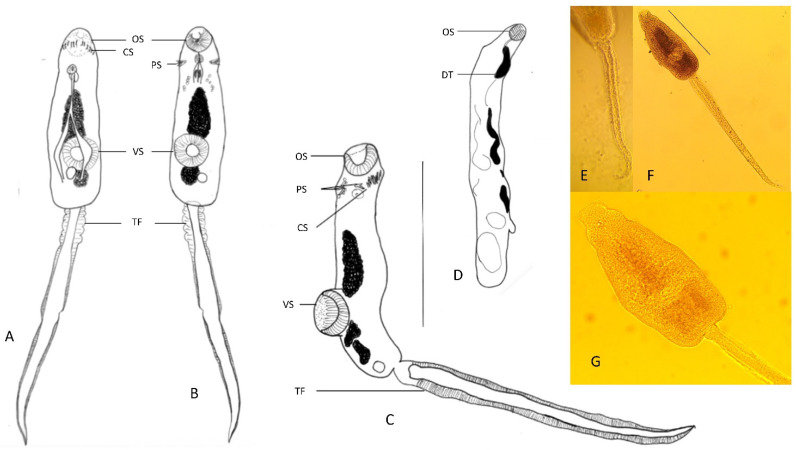
Drawings and photographs of cercaria and redia of *Petasiger* sp. collected from *Isidorella hainesii* examined in this study. (**A**) Dorsal view of whole cercaria. (**B**) Ventral view of whole cercaria. (**C**) Lateral view of whole cercaria. (**D**) Redia. (**E**) Tail of cercaria, showing lateral fins. (**F**) Whole cercaria. (**G**) Cercaria of *Petasiger* sp. showing the granules just posterior to the oral sucker (scale bars: 250 μm).

**Figure 4:**
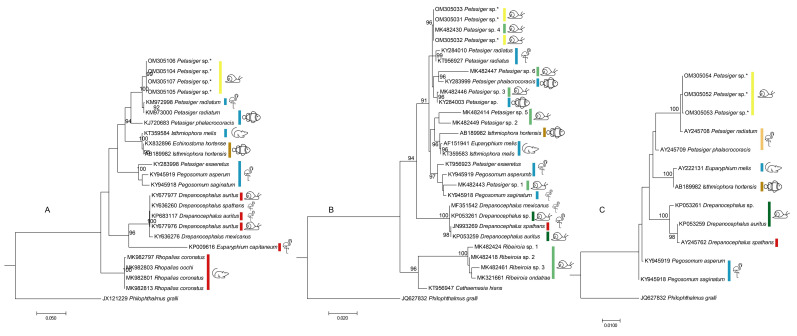
Phylogenetic trees showing the relationship between morphotype C (GenBank accession numbers: OM305031-OM305033, OM305052-OM305054, and OM305104-OM305107) in the present study (indicated with *) with closely related taxa in GenBank for (**A**) ITS2, (**B**) 28S, and (**C**) 18S. Geographical area of collection of specimen indicated by a colored bar (red, North America (USA and Mexico); blue, Europe; yellow, Australia; green, Brazil; brown, Japan and China; light brown, Israel; light green, Rwanda). The host groups that the parasite was recovered from are shown as icons (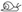, snails; 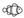, fishes; 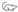, mammals other than bats; 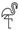, fish-eating birds). The hosts are those listed in Table 1 and include hosts from which parasites/sequences were obtained. Some of these hosts are intermediate/paratenic and some are definitive hosts.

Group C featured cercaria and redia with distinguishing characteristics (Figure 3), including a collar of spines, a shouldered body shape (instead of completely oval), a relatively long tail, and a larger ventral sucker in comparison to its oral sucker. The samples that are referred to as morphotype C in this study were not in a good enough condition to identify the number of collar spines. However, it was possible to see one group of four corner/posterior spines on each side of the oral sucker posteriorly. The specimens all had obvious fins along the tail. They had a total body length and width of 773.13 (705–855) and 332.14 (255–380) µm, respectively (*n* = 14 cercaria). Body length (excluding tail length) was 332.14 (255–380) µm. The tail was 442.50 (385–500) long. Tail width, with and without wing, was 43.75 (40–57.5) and 27.86 (15–40), respectively. Oral and ventral suckers had diameters of 48.75 (40–60) and 69.81 (37.5–85), respectively. Additionally, a small group (2–3) of large granules were obvious posterior to the oral sucker in some specimens. Due to the presence of the collar spines, the cercaria were identified as members of the superfamily Echinostomatiodea [63]. They were identified as belonging to the genus *Petasiger* based on their sequence data (Figure 4). Morphotype C, which was identified as *Petasiger* sp., belongs to the suborder Echinostomata, whereas group A and morphotype B, i.e., *Plagiorchis* and *Choanocotyle hobbsi*, taxonomically belong closer to the suborder Xiphidiata. To avoid producing very large trees, separate phylogenetic trees were created for morphotype C. Sequences from this study were consistently grouped with *Petasiger radiatum*, collected from cormorants in Hungary (Figure 4).

Despite some intraspecific variation among 18S sequences belonging to *C. hobbsi*, the grouping of the sequences of taxa included in all three trees suggests that ITS2, 28S, and 18S are suitable for differentiation between digenean parasites. The phylogenetic tree for members of the superfamily Plagiorchioidea, including group A and morphotype B (Figure 2), also shows Australian taxa group separately from the taxa found in other parts of the world; however, for members of the superfamily Echinostomatoidea, including morphotype C, such distinction was not observed.

## 4. Discussion

Of the snails collected and examined in the present study, *Bullastra lessoni* and *Isidorella hainesii* are native species, whereas *Haitia acuta* is an introduced species. *Choanocotyle hobbsi*, also found in the present study, is a native parasite, which has been recently reported in *Isidorella hainesii* [13]. Herein, we report this native parasite in an introduced snail, *Haitia acuta*, from both natural and aquaculture ponds. This is a case of parasite spillback where a parasite of native hosts infects an invasive host, leading to increased opportunities to infect native species [64]. In a previous study [11], researchers showed that there were only three reports of *H. acuta* shedding larval trematodes (cercariae) within its invasive range in Europe and the Middle East. However, due to a lack of genetic data for parasite larvae, they could not determine the origin of infection of invasive *H. acuta* (i.e., spillback versus spillover). As suggested by Ebbs et al. [11], including parasite genetic data, such as in the present study, is required to better understand the invasion dynamics. Parasite spillback from introduced species could potentially affect all host species in a parasite’s life cycle and cause disease emergence [65]. *Choanocotyle hobbsi* is a parasite of freshwater turtles, many species of which are known to have had a massive decline in their population [66]. However, despite its significance, parasite spillback has been seriously neglected in the conservation plans of the ecologically fragile Murray Darling Basin in Australia. This should be brought to the attention of decision makers and conservation scientists in Australia, considering that over time, as invasive *H. acuta* populations increase, their role in local parasite transmission will also increase.

Parasite spillback might be a common occurrence in this region. Previously, a native nematode parasite, *Contracaecum bancrofti*, was found in several introduced fish hosts, *Carassius auratus*, *Misgurnus anguillicaudatus*, *Cyprinus carpio*, and *Gambusia holbrooki* [67,68]. Understanding the extent of parasite transmission between native and introduced species in the Murray Darling Basin is an important area for future research.

Another parasite found in the present study was *Plagiorchis* sp. found in *Bullastra lessoni*. We did not find an exact genetic match, nor fully developed cercaria, and therefore could not identify it to species level. The parasite belongs to the family Plagiorchiidae (Lühe, 1901), which is a very large family of digenean trematodes. *Plagiorchis* spp. parasitize the digestive system of many species of vertebrates, including humans [53,55,69,70]. In Australia, *P. maculosus* was reported in birds, including *Hirundo neoxena*, *Rhipidura leucophrys*, *R. flabellifera*, *Gymnorhina hypoleuca*, and *Pomatostomus superrciliosus.* Adult Plagiorchiids can be found in any part of the digestive system and can migrate throughout the digestive system of the vertebrate definitive host [55]. Although it is a large group of potentially dangerous parasites for many species, their taxonomy is poorly understood and in need of revision. There are currently 140 described species within the family, making it the largest family of digeneans [55]. Additionally, Johnston and Angel [71] studied the life history of *Plagiorchis jaenschi* and experimentally infected *B. lessoni* (= *Lymnaea lessoni*) with eggs collected from worms from a water rat in South Australia. They also reported a natural infection in the same species of snail.

Lymnaeid snails are known to be the intermediate host for Plagiorchiids [72]. In Angel’s (1959) study, 2/55 snails were found to be infected with small cercaria. Mosquito larvae were experimentally infected with these cercaria and then fed to chickens once they developed into adult mosquitos. Two of the experimentally infected chickens were infected with adult trematodes of *Plagiorchis maculosus.* The eggs from these adult flukes were then successfully used to infect lab-raised snails. Sporocysts and some free cercaria were found in these snails. In the present study, snails were found naturally infected with *Plagiorchis* sp. Because no fully developed cercaria were found, it was not possible to compare the two species morphologically, and Angel [72] did not have genetic data available. It is important to note that many dipteran larvae were found living inside of the *B. lessoni* snail’s shells, with 19 living inside of the infected snail. It is possible that this is how these larvae become infected with *Plagiorchis*. Observationally, many small adult midge-type flies were found in the present study after a few days of keeping the snails, possibly from these dipteran larvae. In future studies, it would be worth catching and identifying these flies and checking them for *Plagiorchis* spp. Additionally, a larger number of lymnaeid snails need to be collected from the same sampling site again in the future, and snails should be kept alive until cercaria are fully developed and are shed into water for the morphology to be completed.

Another parasite found in the present study is *Petasiger* sp. Members of this genus are known to be cosmopolitan and to be found in snails belonging to the family Planorbidae as cercariae, in the esophagus or pharynx of freshwater teleosts as metacercariae, and in the intestine of fish-eating birds (Anhingidae, Phalacrocoracidae, Phoenicopteridae, Podicipedidae, and occasionally Anatidae, and Laridae) in the adult form [73]. Few species of *Petasiger* have been reported from Australian birds [74], with *P. australis* reported from grebes in South Australia [71], *P. exaeretus* from cormorants and shags in South Australia, NSW, and Queensland, although not from the Murrumbidgee catchment area [75], and a *Petasiger* sp. from a barn owl in South Australia [74]. Johnston and Angel [71] described a cercaria (*Cercaria gigantura*), presumed to be the larval stage of *P. australis*, to have a total of 19 collar spines and a “relatively huge tail” that affected the swimming motion of the cercaria. A comparison of the measurements presented for *C. gigantura* with the cercaria collected in this study showed that although the tail lengths were approximately equal, the body length for *C. gigantura* was shorter (105–267 μm) compared to the cercaria collected in this study. Both *P. exaeretus* and the *Petasiger* sp., however, have 27 collar spines; this former species has also been reported from cormorants from Europe and Japan [75]. As there are no genetic sequences for adult specimens of *Petasiger* spp. collected in Australia for comparison, whether the *Petasiger* sp. collected in this study is the larval stage of one of the previously described species or is a new, undescribed species cannot yet be determined.

In the present study, *Petasiger* sp. could not be identified to species level due to the absence of any identical and comparable sequence data from adult specimens. The cercaria found in our study had similar morphology to those reported by Našincová et al. [76], including similarly located posterior and collar spines; however, the staining procedure in our study did not allow for a clear enough visualization of the exact number of collar spines present. Additionally, some of the cercaria collected in our study possessed a small group of large granules posterior to the oral sucker, similar to that described by Laidemitt et al. [53] for *Petasiger* sp. 3 and sp. 4, collected from snails in Kenya. The results of the 28S analysis found the sequences collected in this study to be very close to those for *Petasiger* sp. 4 (Figure 4B). In the tree presented by Laidemitt, Brant, Mutuku, Mkoji, and Loker [53], *Petasiger* sp. 4 matched an adult worm collected from *Microcarbo africanus* in Kenya and was grouped with an undescribed *Echinostoma* sp., collected in Australia by Morgan and Blair [77]. *Petasiger* sp. 4 possessed 27 collar spines [53], whereas the undescribed *Echinostoma* sp. possessed over 40 collar spines [77]; the number of collar spines could not be determined in the specimens collected in this study, potentially due to their young stage of development and being dissected from the snails.

When studying *P. radiatus*, Našincová, Scholz, and Moravec [76] did not find sporocysts in any of the naturally or experimentally infected snails, but rediae were found in both, similar to our results. In Europe, the cercarial stage of *Petasiger* has been found in freshwater pulmonate snails *Gyraulus albus* and *Segmentina nitida*, both of which belong to the family Planorbidae, and *Radix auricularia*, a pulmonate Lymanaeid [76]. In our study, the cercarial stage was found in *Isidorella hainesii*, a native Australian snail, also from the family Planorbidae. Pulmonates have air sacs to enable them to breathe air, meaning they must go to the surface of the water from time to time. This could explain why the cercaria of many *Petasiger* spp. have long tails with fins, as they must move through the water to find snails that may be near the surface of the water. The *Petasiger* sp. cercaria found in the present study had these morphological characteristics and were also observed to be highly motile for a number of hours after exiting the snail host.

In the study by Našincová, Scholz, and Moravec [76], experimentally infected fish had metacercaria encysted around the mouth and gills, eyes, nasal hollows, and in the skin. Metacercaria from the Echinostomatidae family are frequently found in fish and, close to where snails were collected in the present study, various fish were found to be infected with metacercaria of Trematoda [78,79]. However, they did not belong to *Petasiger* sp. Therefore, it is important for parasites found in wild and farmed fish to be examined properly for specific identification and to inform subsequent management decisions. *Petasiger* spp. are a commonly found trematode parasite in the intestine of piscivorous birds (particularly cormorants) in Europe, Asia, and Africa [48,76]. In Australia, *Petasiger australis* has been reported from Hoary-headed Grebe, *Poliocephalus poliocephalus* [71].

Aquaculture ponds are known to favor populations of predators that could be potential definitive hosts, such as aquatic birds [80]. Although our sampling sites were from both natural reservoirs and aquaculture farms, due to significant differences in the number of snails collected, no reliable conclusion can be drawn about any significant difference in the population of the infected snails between different sites. An interesting area for future study would be to investigate this matter.

## 5. Conclusions

The knowledge of parasites in Australian wildlife is poor, with most host species, especially those that act as intermediate hosts, unstudied. The documentation of this fauna, including both morphological and molecular characterization, is important to ensure an understanding of biodiversity, parasite transmission, and ecosystem impacts.

## Figures and Tables

**Table 1:** List of sequences used for building phylogenetic trees. Sequences are arranged in alphabetical order of Trematode species.

Trematode Species	Trematode Family	GenBank Accession No	Host	Parasite Development Stage	Locality	Reference
*Alloglossidium anomaphagis*	Alloglossidiidae	MH041376	*Daphnia obtusa*	Adult	USA	[19]
*Alloglossidium floridense*	Alloglossidiidae	MH041390	*Noturus gyrinus*	Adult	USA	[19]
*Alloglossidium fonti*	Alloglossidiidae	MH041395	*Ameiurus melas*	Adult	USA	[19]
*Alloglossidium greeri*	Alloglossidiidae	MH041387	*Cambarellus shufeldtii*	Adult	USA	[19]
*Alloglossidium hamrumi*	Alloglossidiidae	MH041415	*Macrobdella decora*	Adult	USA	[19]
*Alloglossidium hirudicola*	Alloglossidiidae	MH041418	*Macrobdella decora*	Adult	USA	[19]
*Alloglossidium kenti*	Alloglossidiidae	MH041405	*Ictalurus punctatus*	Adult	USA	[19]
*Alloglossidium macrobdellensis*	Alloglossidiidae	MH041413	*Macrobdella decora*	Adult	USA	[19]
*Alloglossidium progeneticum*	Alloglossidiidae	MH041382	*Procambarus spiculifer*	Adult	USA	[19]
*Alloglossidium renale*	Alloglossidiidae	MH041385	*Palaemonetes kadiakensis*	Adult	USA	[19]
*Alloglossidium schmidti*	Alloglossidiidae	MH041419	*Haemopis grandis*	Adult	Canada	[19]
*Alloglossidium turnbulli*	Alloglossidiidae	MH041423	*Haemopis grandis*	Adult	USA	[19]
*Aptorchis aequalis*	Plagiorchiidae	EU334369	*Emydura krefftii*	Adult	Australia	[20]
*Aptorchis glandularis*	Plagiorchiidae	EU334368	*Emydura australis*	Adult	Australia	[20]
*Aptorchis kuchlingi*	Plagiorchiidae	HQ680841, HQ680845	*Chelodina oblonga*	Adult	Australia	[21]
*Aptorchis megacetabulus*	Plagiorchiidae	EF014730	*Chelodina rugosa*	Adult	Australia	[22]
*Aptorchis megapharynx*	Plagiorchiidae	EF014727	*Chelodina longicollis*	Adult	Australia	[22]
*Aptorchis pearsoni*	Plagiorchiidae	EF014728	*Chelodina expansa*	Adult	Australia	[22]
*Auridostomum chelydrae*	Auridistomidae	AY222159	*Chelydra serpentina*	Adult	USA	[23]
*Brachycladium goliath* (OUTGROUP)	Brachycladiidae	KR703279	*Balaenoptera acutorostrata*	Adult	UK	[24]
*Brachycoelium salamandrae*	Brachycoeliidae	AY222160	*Salamandra salamandra*	Adult	Ukraine	[23]
*Cathaemasia hians*	Echinostomatidae	KT956947	*Planorbis planorbis*	Cercaria	Czech Republic	[25]
*Cephalogonimus retusus*	Cephalogonimidae	AJ287489	*Rana ridibunda*	Adult	-	[26]
*Choanocotyle* *hobbsi*	Choanocotylidae	EU196356	*Chelodina oblonga*	Adult	Australia	[27]
*Choanocotyle hobbsi*	Choanocotylidae	MW682817-MW682819	*Isidorella hainesii*	Cercaria	Australia	[13]
*Choanocotyle hobbsi*	Choanocotylidae	MW684083-MW684089	*Isidorella hainesii*	Cercaria	Australia	[13]
*Choanocotyle hobbsi*	Choanocotylidae	MW686389, MW686392-MW686393	*Isidorella hainesii*	Cercaria	Australia	[13]
*Choanocotyle hobbsi*	Choanocotylidae	OM305034-OM305039	*Haitia acuta*	Cercaria	Australia	This study
*Choanocotyle hobbsi*	Choanocotylidae	OM305043-OM305048	*Haitia acuta*	Cercaria	Australia	This study
*Choanocotyle hobbsi*	Choanocotylidae	OM305095-OM305100	*Haitia acuta*	Cercaria	Australia	This study
*Choanocotyle nematoides*	Choanocotylidae	AY116862-AY116864, AY116867	*Chelodina oblonga*	Adult	Australia	[28]
*Choanocotyle nematoides*	Choanocotylidae	EU196357-EU196358	*Emydura krefftii*	Adult	Australia	[27]
*Choanocotyle nematoides*	Choanocotylidae	EU196359-EU196360	*Emydura macquarii*	Adult	Australia	[27]
*Choanocotyle platti*	Choanocotylidae	EU196355	*Chelodina rugosa*	Adult	Australia	[27]
*Choledocystus hepatica*	Plagiorchiidae	AY875679	*Rhinella marina*	Adult	Mexico	[29]
*Dasymetra nicolli*	Reniferidae	AF433672	*Nerodia rhombifer*	Adult	USA	[30]
*Drepanocephalus auritus*	Echinostomatidae	KP053259	*Biomphalaria stramina*	Cercaria	Brazil	[31]
*Drepanocephalus auritus*	Echinostomatidae	KP683117	*Phalacrocorax auritus*	Adult	USA	[32]
*Drepanocephalus auritus*	Echinostomatidae	KY677976, KY677977	*Biomphalaria havanensis*	Cercaria	USA	[33]
*Drepanocephalus mexicanus*	Echinostomatidae	KY636276	*Nannopterum brasilianus*	Adult	-	[34]
*Drepanocephalus mexicanus*	Echinostomatidae	MF351542	*Nannopterum brasilianus*	Adult	-	[34]
*Drepanocephalus* sp.	Echinostomatidae	KP053261	*Biomphalaria stramina*	Cercaria	Brazil	[31]
*Drepanocephalus spathans*	Echinostomatidae	AY245762	Not stated	Not stated	Not stated	Unpublished
*Drepanocephalus spathans*	Echinostomatidae	JN993269	*Phalacrocorax auritus*	Adult	USA	[35]
*Drepanocephalus spathans*	Echinostomatidae	KY636260	*Nannopterum brasilianus*	Adult	-	[34]
*Echinostoma hortense ^a^*	Echinostomatidae	KX832896	*Misgurnus anguillicaudatus*	Metacercariae	China	[36]
*Euparyphium capitaneum*	Echinostomatidae	KP009616	*Anhinga anhinga*	Adult	USA	[37]
*Euparyphium melis ^b^*	Echinostomatidae	AF151941	*Nyctereutes procyonoides*	Adult	Ukraine	[38]
*Euparyphium melis ^b^*	Echinostomatidae	AY222131	*Nyctereutes procyonoides*	Adult	Ukraine	[23]
*Glypthelmins africana*	Glypthelminthidae	OL413039	*Hyperolius viridiflavus*	Adult	Rwanda	[39]
*Glypthelmins quieta*	Glypthelminthidae	AJ287517	*Rana catesbeiana*	Adult	-	[26]
*Haematoleochus longiplexus*	Haematoleochidae	AJ287520	*Rana catesbeiana*	Adult	-	[26]
*Haematoleochus* sp.	Haematoleochidae	MH285261	*Odorrana grahami*	Adult	China	Unpublished
*Haplometra cylindracea*	Plagiorchiidae	AF151933	*Rana arvalis*	Adult	Ukraine	[38]
*Haplometroides intercaecalis*	Plagiorchiidae	MH206169	*Phalotris matogrossensis*	Adult	Brazil	[40]
*Isthmiophora hortensis*	Echinostomatidae	AB189982	*Misgurnus anguillicaudatus*	Adult	Japan	[41]
*Isthmiophora melis*	Echinostomatidae	KT359583-KT359584	*Apodemus agrariu*	Adult	Poland	[42]
*Lechriorchis tygarti*	Reniferidae	JF820599-JF62600	*Lithobates sylvaticus*	Metacercaria	USA	[43]
*Macroderoides typicus*	Macroderoididae	AY222158	*Lepisosteus platostomus*	Adult	USA	[23]
*Magnivitellinum simplex*	Alloglossidiidae	KU535678, KU535681-KU535683	*Astyanax mexicanus*	Adult	Mexico	[44]
*Mesocoelium lanfrediae*	Brachycoeliidae	JQ886404	*Rhinella marina*	Adult	Brazil	[45]
*Neoglyphe locellus*	Omphalometridae	AF300330	*Sorex araneus*	Adult	Ukraine	[30]
*Neoglyphe sobolesi*	Omphalometridae	AF300329	*Sorex araneus*	Adult	Ukraine	[30]
*Omphalometra flexuosa*	Omphalometridae	AF300333	*Planorbis planorbis*	Cercaria	Poland	[30]
*Opisthioglyphe ranae*	Telorchiidae	AF151929	*Rana arvalis*	Adult	Ukraine	[38]
*Opisthioglyphe ranae*	Telorchiidae	AY222157	*Rana arvalis*	Adult	Ukraine	[23]
*Opisthioglyphe ranae*	Telorchiidae	MK585340-MK585341	*Pelophylax ridibundus*	Metacercaria	Russia	Unpublished
*Paryphostomum radiatum ^c^*	Echinostomatidae	KM972998, KM973000	*Phalacrocorax carbo*	Adult	Hungary	[46]
*Paryphystomum radiatium ^c^*	Echinostomatidae	AY245708	*Phalacrocorax carbo*	Adult	Israel	[47]
*Pegosomum asperum*	Echinostomatidae	KY945919	*Ardea alba*	Adult	Germany	Unpublished
*Pegosomum saginatum*	Echinostomatidae	KY945918	*Ardea alba*	Adult	Germany	Unpublished
*Petasiger exaeretus*	Echinostomatidae	KT956923	*Phalacrocorax carbo*	Adult	Ukraine	[25]
*Petasiger exaeretus*	Echinostomatidae	KY283998	*Phalacrocorax carbo*	Adult	Hungary	[48]
*Petasiger phalacrocoracis*	Echinostomatidae	AY245709	*Phalacrocorax carbo*	Adult	Israel	[47]
*Petasiger phalacrocoracis*	Echinostomatidae	KJ720683	*Rutilus rutilus*	Metacercaria	Hungary	[46]
*Petasiger phalacrocoracis*	Echinostomatidae	KY283999	*Rutilus rutilus*	Metacercaria	Hungary	[49]
*Petasiger radiatus*	Echinostomatidae	KJ956927	*Phalacrocorax carbo*	Adult	Ukraine	[25]
*Petasiger radiatus*	Echinostomatidae	KY284010	*Phalacrocorax carbo*	Adult	Hungary	[49]
*Petasiger* sp.	Echinostomatidae	KY284003	*Rutilus rutilus*	Metacercaria	Hungary	[49]
*Petasiger* sp.	Echinostomatidae	OM305031-OM305033	*Isidorella hainesii*	Cercaria	Australia	This study
*Petasiger* sp.	Echinostomatidae	OM305052-OM305054	*Isidorella hainesii*	Cercaria	Australia	This study
*Petasiger* sp.	Echinostomatidae	OM305104-OM305107	*Isidorella hainesii*	Cercaria	Australia	This study
*Petasiger* sp. 1	Echinostomatidae	MK482443	*Radix natalensis*	Cercaria	Kenya	[50]
*Petasiger* sp. 2	Echinostomatidae	MK482449	*Bulinus globosus*	Cercaria	Kenya	[50]
*Petasiger* sp. 3	Echinostomatidae	MK482446	*Radix natalensis*	Cercaria	Kenya	[50]
*Petasiger* sp. 4	Echinostomatidae	MK482430	*Biomphalaria pfeifferi*	Cercaria	Kenya	[50]
*Petasiger* sp. 5	Echinostomatidae	MK482414	*Bulinus* sp.	Cercaria	Kenya	[50]
*Petasiger* sp. 6	Echinostomatidae	MK482447	*Bulinus* sp.	Cercaria	Kenya	[50]
*Philophthalmus gralli* (OUTGROUP)	Philophthalmidaae	JQ627832	*Tachuris rubrigastra*	Adult	Peru	[51]
*Philophthalmus gralli* (OUTGROUP)	Philophthalmidaae	JX121229	*Tachuris rubrigastra*	Adult	Peru	[52]
*Plagiorchis* *elegans*	Plagiorchiidae	KF556678	*Lymnaea stagnalis*	Cercaria	USA	[35]
*Plagiorchis* *elegans*	Plagiorchiidae	KJ533393	*Lymnaea stagnalis*	Cercaria	Czech Republic	[53]
*Plagiorchis* *elegans*	Plagiorchiidae	MW001064, MW001068	*Lymnaea stagnalis*	Cercaria	Denmark	[54]
*Plagiorchis koreanus*	Plagiorchiidae	AF151930	*Nyctalus noctula*	Adult	Ukraine	[38]
*Plagiorchis maculosus*	Plagiorchiidae	KJ533395	*Lymnaea stagnalis*	Cercaria	Czech Republic	[53]
*Plagiorchis maculosus*	Plagiorchiidae	MK641807	*Hirundo rustica*	Adult	Pakistan	[55]
*Plagiorchis maculosus*	Plagiorchiidae	MW001083	*Lymnaea stagnalis*	Cercaria	Denmark	[54]
*Plagiorchis neomidis*	Plagiorchiidae	KJ533397	*Lymnaea stagnalis*	Cercaria	Czech Republic	[53]
*Plagiorchis* sp.	Plagiorchiidae	KJ533398	*Lymnaea stagnalis*	Cercaria	Czech Republic	[53]
*Plagiorchis* sp.	Plagiorchiidae	MW001088	*Lymnaea stagnalis*	Cercaria	Denmark	[54]
*Plagiorchis* sp.	Plagiorchiidae	MW001090	*Stagnicola palustris*	Cercaria	Denmark	[54]
*Plagiorchis* sp.	Plagiorchiidae	MW001091	*Ampullaceana balthica*	Cercaria	Denmark	[54]
*Plagiorchis* sp.	Plagiorchiidae	MW001113	*Lymnaea stagnalis*	Cercaria	Denmark	[54]
*Plagiorchis* sp.	Plagiorchiidae	OM305040-OM305042	*Bullastra lessoni*	Cercaria and Sporocysts	Australia	This study
*Plagiorchis* sp.	Plagiorchiidae	OM305049-OM305050	*Bullastra lessoni*	Cercaria and Sporocysts	Australia	This study
*Plagiorchis* sp.	Plagiorchiidae	OM305101-OM305103	*Bullastra lessoni*	Cercaria and Sporocysts	Australia	This study
*Plagiorchis* sp. 1	Plagiorchiidae	KX160477	*Hydropsyche* sp.	Metacercaria	Germany	[56]
*Plagiorchis* sp. 1	Plagiorchiidae	MW528604	*Ampullaceana balthica*	Cercaria	Iceland	[57]
*Plagiorchis* sp. 2	Plagiorchiidae	MW001092	*Stagnicola palustris*	Cercaria	Denmark	[54]
*Plagiorchis* sp. 2	Plagiorchiidae	MW528605	*Radix balthica*	Cercaria	Iceland	[57]
*Plagiorchis* sp. 3	Plagiorchiidae	KX160474	*Lepidostematus* sp.	Metacercaria	Germany	[56]
*Plagiorchis* sp. 3	Plagiorchiidae	MW528606	*Radix balthica*	Cercaria	Ireland	[57]
*Plagiorchis* sp. 5	Plagiorchiidae	MW528611	*Radix balthica*	Cercaria	Ireland	[57]
*Plagiorchis* sp. 7	Plagiorchiidae	MW528616	*Radix balthica*	Cercaria	Ireland	[57]
*Plagiorchis* sp. 8	Plagiorchiidae	MW528619	*Radix balthica*	Cercaria	Ireland	[57]
*Plagiorchis* sp. 9	Plagiorchiidae	MW528621	*Stagnicola fuscus*	Cercaria	Ireland	[57]
*Plagiorchis* sp. A	Plagiorchiidae	LC599522	*Radix auricularia*	Daughter Sporocyst	Japan	[58]
*Plagiorchis* sp. B	Plagiorchiidae	LC599524	*Radix auricularia*	Daughter Sporocyst	Japan	[58]
*Plagiorchis* sp. C	Plagiorchiidae	LC599525	*Radix auricularia*	Daughter Sporocyst	Japan	[58]
*Plagiorchis* sp. Lineage 1	Plagiorchiidae	MW528622	*Stagnicola elodes*	Cercaria	USA	[57]
*Plagiorchis* sp. Lineage 4	Plagiorchiidae	MW528623	*Stagnicola elodes*	Cercaria	USA	[57]
*Plagiorchis* sp. Lineage 6	Plagiorchiidae	MW528624	*Stagnicola elodes*	Cercaria	USA	[57]
*Plagiorchis* sp. Lineage 9	Plagiorchiidae	MW528626	*Stagnicola elodes*	Cercaria	USA	[57]
*Plagiorchis vespertilionis*	Plagiorchiidae	AF151931	*Myotis daubentoni*	Adult	Ukraine	[38]
*Renifer aniarum*	Reniferidae	HQ665459	*Nerodia rhombifer*	Adult	USA	[59]
*Renifer kansensis*	Reniferidae	LC557508, LC557512	*Elaphe quadrivirgata*	Adult	Japan	[60]
*Rhopalias coronatus*	Echinostomatidae	MK982797, MK982801, MK982813	*Didelphismarsupialis virginiana*	Adult	Mexico	[61]
*Rhopalias oochi*	Echinostomatidae	MK982803	*Didelphismarsupialis marsupialis*	Adult	Mexico	[61]
*Ribeiroia ondatrae*	Echinostomatidae	MK321661	*Biomphalaria sudanica*	Cercaria	Kenya	[50]
*Ribeiroia* sp. 1	Echinostomatidae	MK482424	*Biomphalaria sudanica*	Cercaria	Kenya	[50]
*Ribeiroia* sp. 2	Echinostomatidae	MK482418	*Biomphalaria sudanica*	Cercaria	Kenya	[50]
*Ribeiroia* sp. 3	Echinostomatidae	MK482461	*Biomphalaria sudanica*	Cercaria	Kenya	[50]
*Rubenstrema exasperatum*	Omphalometridae	AF300331	*Sorex araneus*	Adult	Ukraine	[30]
*Rubenstrema exasperatum*	Omphalometridae	AJ287572	*Crocidura leucodon*	-	-	[26]
*Rubenstrema exasperatum*	Omphalometridae	MK585231	*Planorbarius corneus*	Metacercaria	Russia	Unpublished
*Sigmapera cincta*	Plagiorchiidae	EF411200	*Emydura kreffti*	Not stated	Australia	Unpublished
*Skrjabinoeces similis*	Plagiorchiidae	AJ287575	*Rana ridibunda*	-	-	[26]
*Skrjabinoeces similis*	Plagiorchiidae	AY222279	*Pelophylax ridibundus*	Adult	Bulgaria	[23]
*Telorchis assula*	Telorchiidae	AF151915	*Natrix natrix*	Adult	Ukraine	[38]
*Telorchis assula*	Telorchiidae	AY222156	*Natrix natrix*	Adult	Ukraine	[23]
*Telorchis bonnerensis*	Telorchiidae	JF820591	*Ambystoma tigrinum*	Adult	USA	[43]
*Telorchis bonnerensis*	Telorchiidae	JF820593	*Lithobates sylvaticus*	Metacercaria	USA	[43]
*Telorchis* sp.	Telorchiidae	OL960085	*Planorbella trivolvis*	Not stated	USA	

*^a^* Sequence listed under *Echinostoma hortense*, although species had been transferred to the genus *Isthmiophora* by Ref. [62]; *^b^* Sequence wrongly listed as *Euparyphium melis*; species is within the genus *Isthmiophora*, see Ref. [62]; *^c^* Sequence listed under *Paryphostomum radiatum*; species has subsequently been transferred to the genus *Petasiger* by Tkach, Kudlai and Kostadinova [24].

**Table 2:** Snails examined in the present study and the parasites found. Locality data refer to the location numbers identified in Figure 1.

Snail Species	No. Examined (No. Infected)	Locality	Provisional Parasite Identification (Groups/Morphotype)	Parasite Species Found	Infected Snail Code	No. of Sporocysts	No. of Redia	No. of Cercaria	Genetic ID (Y/N)
*Bullastra lessoni*	11 (1)	1	A	*Plagiorchis* sp.	11	>100	0	>100	Y
*Haitia acuta*	88 (4)	2	B	*Choanocotyle hobbsi*	47, 123, 124, 126	0, 0, 0, 0	0, 0, 0, 0	5, 1, 1, 2	N
	11 (0)	4	-	-	-	-	-	-	-
	8 (1)	3	B	*Choanocotyle hobbsi*	34	10–50	0	50–100	Y
*Isidorella hainesii*	150 (2)	2	C	*Petasiger* sp.	94, 85	0	>100	50–100	Y
	4 (0)	4	-	-	-	-	-	-	-
	3 (0)	3	-	-	-	-	-	-	-

## Data Availability

Not applicable.
